# Human Papillomavirus (HPV) Upregulates the Cellular Deubiquitinase UCHL1 to Suppress the Keratinocyte's Innate Immune Response

**DOI:** 10.1371/journal.ppat.1003384

**Published:** 2013-05-23

**Authors:** Rezaul Karim, Bart Tummers, Craig Meyers, Jennifer L. Biryukov, Samina Alam, Claude Backendorf, Veena Jha, Rienk Offringa, Gert-Jan B. van Ommen, Cornelis J. M. Melief, Daniele Guardavaccaro, Judith M. Boer, Sjoerd H. van der Burg

**Affiliations:** 1 Center for Human and Clinical Genetics, Leiden University Medical Center, Leiden, The Netherlands; 2 Department of Immunohematology and Blood Transfusion, Leiden University Medical Center, Leiden, The Netherlands; 3 Department of Clinical Oncology, Leiden University Medical Center, Leiden, The Netherlands; 4 Department of Microbiology and Immunology, The Pennsylvania State University College of Medicine, Hershey, Pennsylvania, United States of America; 5 Laboratory of Molecular Genetics, Leiden Institute of Chemistry, Gorlaeus Laboratories, Leiden University, Leiden, The Netherlands; 6 Hubrecht Institute-KNAW and University Medical Center Utrecht, Utrecht, The Netherlands; Vaccine & Gene Therapy Institute of Florida, United States of America

## Abstract

Persistent infection of basal keratinocytes with high-risk human papillomavirus (hrHPV) may cause cancer. Keratinocytes are equipped with different pattern recognition receptors (PRRs) but hrHPV has developed ways to dampen their signals resulting in minimal inflammation and evasion of host immunity for sustained periods of time. To understand the mechanisms underlying hrHPV's capacity to evade immunity, we studied PRR signaling in non, newly, and persistently hrHPV-infected keratinocytes. We found that active infection with hrHPV hampered the relay of signals downstream of the PRRs to the nucleus, thereby affecting the production of type-I interferon and pro-inflammatory cytokines and chemokines. This suppression was shown to depend on hrHPV-induced expression of the cellular protein ubiquitin carboxyl-terminal hydrolase L1 (UCHL1) in keratinocytes. UCHL1 accomplished this by inhibiting tumor necrosis factor receptor-associated factor 3 (TRAF3) K63 poly-ubiquitination which lead to lower levels of TRAF3 bound to TANK-binding kinase 1 and a reduced phosphorylation of interferon regulatory factor 3. Furthermore, UCHL1 mediated the degradation of the NF-kappa-B essential modulator with as result the suppression of p65 phosphorylation and canonical NF-κB signaling. We conclude that hrHPV exploits the cellular protein UCHL1 to evade host innate immunity by suppressing PRR-induced keratinocyte-mediated production of interferons, cytokines and chemokines, which normally results in the attraction and activation of an adaptive immune response. This identifies UCHL1 as a negative regulator of PRR-induced immune responses and consequently its virus-increased expression as a strategy for hrHPV to persist.

## Author Summary

A persistent infection with high-risk human papillomavirus (hrHPV) may cause cancer. Whereas keratinocytes – the cells infected by hrHPV – are equipped with different receptors allowing them to recognize invading pathogens and to activate the immune system, hrHPV has developed ways to evade the host's immune response for sustained periods of time. We showed that hrHPV accomplishes this by interfering with the signaling of the pathogen receptors, thereby hampering the production of cytokines that are known to attract and activate the immune system. HrHPV accomplishes this by upregulating the expression of a cellular protein called ubiquitin carboxyl-terminal hydrolase L1 (UCHL1). This protein suppresses the activation of signals downstream of the pathogen receptor leading to reduced transcription factor activation and downstream gene expression, in particular that of type I interferon and pro-inflammatory cytokines. This lowers the attraction of immune cells and thereby the chance of hrHPV-infected cells to be recognized and eliminated and as such enables hrHPV to persist.

## Introduction

Human papillomaviruses (HPVs) are absolutely species-specific small double-stranded DNA viruses. Persistent infections with a number of HPVs, predominantly types 16 and 18, can induce cancers of the anogenitalia as well as of the head and neck region. These so-called high-risk HPVs (hrHPVs) are widespread within all human populations where they are commonly transmitted by sexual contact [1]. The undifferentiated keratinocytes of the squamous epithelia are the primary target for hrHPV [2] where it establishes an infection that can last for up to 2 years, indicating that hrHPV has evolved mechanisms to effectively evade the innate and adaptive immune mechanisms protecting the majority of immunocompetent hosts [3], [4].

Viruses and microbes contain pathogen-associated molecular patterns that are recognized by the host's pattern recognition receptors (PRRs), comprising the Toll-like receptors (TLRs), nucleotide oligomerization domain-like receptors and retinoic acid-inducible gene I (RIG-I)-like receptors (RLRs) [5]. While all of these receptors activate signaling cascades that lead to activation of NF-κB via the canonical route, only RLRs and some TLRs activate interferon regulatory factors (IRFs) which induce the production of type I interferons (IFN) and other effector molecules [6]. The signals from the PRR to the cell nucleus are coordinated via ubiquitination, including that of the different tumor-necrosis factor receptor-associated factors (TRAFs) and the NF-κB essential modulator (NEMO). Poly-ubiquitination of TRAF and NEMO allows downstream signaling whereas disassembly of the formed poly-ubiquitin chains by deubiquitinating enzymes provides a mechanism for downregulating immune responses [6], [7].

Keratinocytes (KCs) express TLRs 1–3, TLR5, TLR6, TLR10, RIG-I, protein kinase R (PKR), and MDA5 independent of their differentiation status and gain the expression of TLR9 upon full differentiation indicating that these cells may respond to pathogenic challenges [8], [9], [10]. Thus, KCs should be able to sense the presence of hrHPV genomic DNA directly via TLR9 or indirectly via RIG-I [5], [11], [12]. The expression levels of these PRR were not altered in hrHPV+ KCs [10]. However, via genome-wide expression profiling of keratinocytes activated through TLR3, PKR, RIG-I and MDA-5 we found that the presence of hrHPV dampens a network of genes encoding chemotactic, pro-inflammatory and antimicrobial cytokines suggesting that HPV's immune evasion strategy may rely on countering PRR-mediated cell signaling [10].

To understand the mechanisms underlying hrHPV's capacity to dampen PRR signaling we utilized a system that resembles the natural infection with HPV as closely as possible. It comprises the use of primary KCs that stably maintain the hrHPV genome as episomes following transfection. These hrHPV+ KCs grow at similar rates as non-transfected KC and have been shown to mimic HPV infection *in vivo* as they undergo the entire differentiation-dependent HPV life cycle documented by genome amplification, late gene expression, and virus production, upon culture of hrHPV+ KCs in organotypic raft cultures [13], [14], [15]. In addition, we used non-infected primary KC cultures and primary KCs newly infected with authentic HPV16 virions. These primary KCs were compared with respect to PRR signaling under different conditions and resulted in the identification of the cellular enzyme ubiquitin carboxyl-terminal hydrolase L1 (*UCHL1*) that was specifically upregulated by hrHPV in primary keratinocytes to dampen innate immunity. UCHL1 acted on the PRR-signaling pathway adaptor molecules TRAF3 and NEMO and its inhibition restored PRR-induced production of IFNβ and pro-inflammatory and chemotactic cytokines.

## Results

### High risk HPV is associated with a decreased induction of type I IFN and pro-inflammatory cytokines following stimulation of keratinocytes via different pattern-recognition receptors

**Figure 1:**
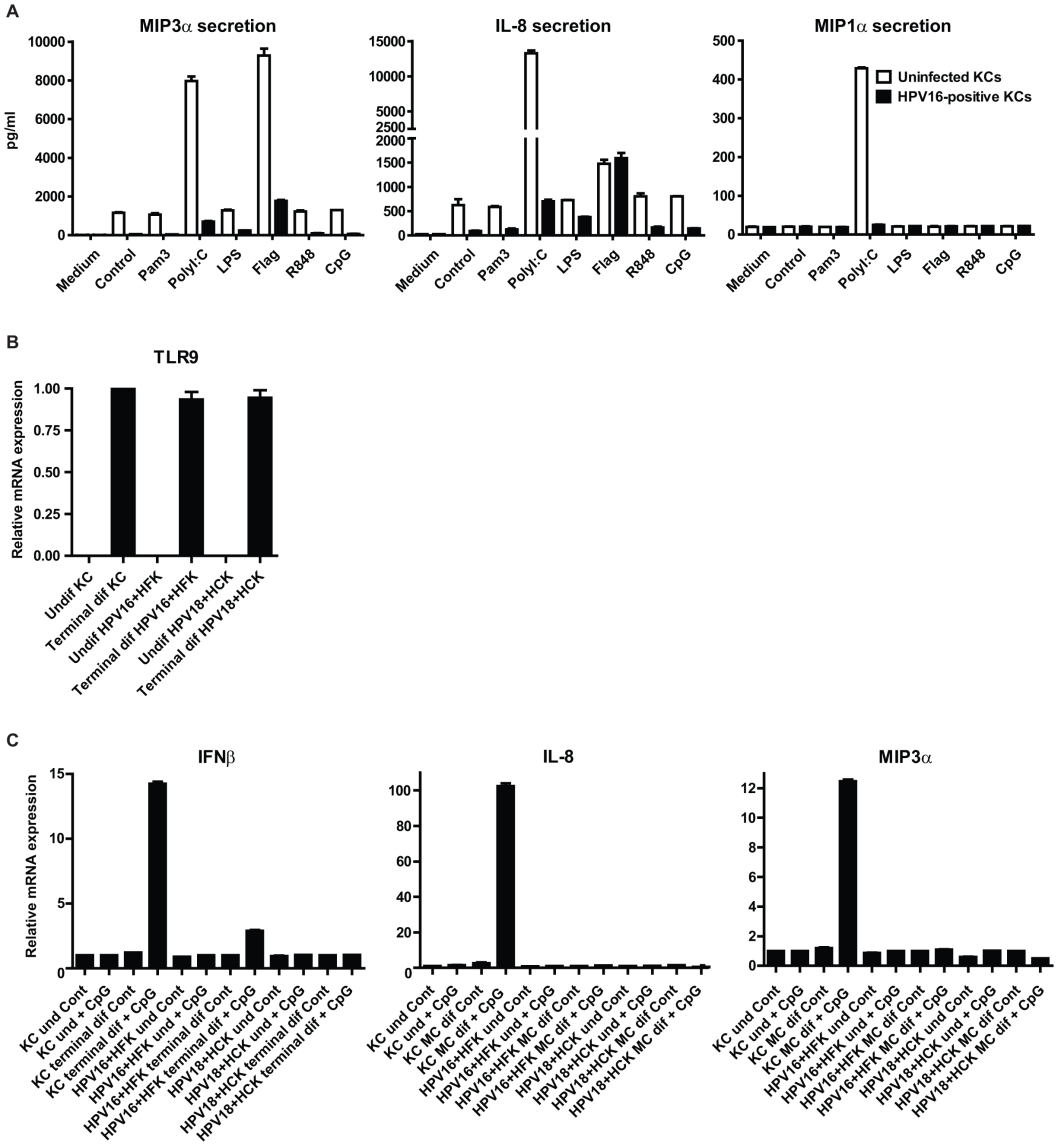
The presence of high risk human papillomavirus interferes with pattern recognition receptor (PRR) signaling of keratinocytes. (**A**) Cytokine production of non-differentiated uninfected or HPV16+ keratinocytes after stimulation with different indicated PRR stimuli as measured by ELISA. (**B**) *TLR9* expression as measured by qRT-PCR on total RNA samples from undifferentiated (und) and terminally differentiated (terminal dif) uninfected KCs, and HPV16 and HPV18 positive KC cultures. (**C**) *IFNβ*, *IL-8* and *MIP3α* expression levels in unstimulated or CpG ODN-stimulated uninfected KCs, and two different HPV (16 or 18) positive KC cultures as examined by qRT-PCR. KCs were either left undifferentiated (und) or terminally differentiated (terminal dif) after which they were stimulated with CpG (10 µg/ml) for 7 hours. (**B**–**C**) Gene expression was normalized using *GAPDH* mRNA expression levels.

Undifferentiated uninfected primary KCs and hrHPV+ KCs were tested for their capacity to respond to triggers of innate immunity by incubation with Pam3CSK4 (TLR1/2), poly(I∶C) (TLR3, RIG-I, PKR and MDA-5) [9], lipopolysaccharide (LPS, TLR4), flagellin (TLR5), R848 (TLR7/8), or CpG (TLR9). The supernatant of non-infected keratinocytes contained higher levels of MIP3α and IL-8 but not MIP1α than hrHPV+ KCs at the basal level. Activation with poly(I∶C) induced the production of high amounts of MIP3α, IL-8 and MIP1α in KCs but not in hrHPV+ KCs. Flagellin especially triggered the production of MIP3α by KCs but not in hrHPV+ KCs, although IL-8 was still produced (Figure 1A). The function of TLR9, expressed only at high protein levels in differentiated keratinocytes as measured by immunohistochemistry [10] and by RT-qPCR (Figure 1B), was tested by the capacity of CpG oligodeoxynucleotides (CpG ODN) to trigger the expression of mRNAs of pro-inflammatory cytokines and chemokines. Because suspension in methyl cellulose – to differentiate keratinocytes – does not allow the harvest of supernatant, secreted protein levels could not be measured. However, the experiments clearly showed that CpG ODN-stimulation resulted in the gene expression of *IFNB1* (*IFNβ*), *IL-8* and *CCL20* (*MIP3α*) in differentiated KCs but not in undifferentiated KC cultures (Figure 1C). As a control, KCs were also stimulated with poly(I∶C) as TLR3, RIG-I and MDA-5 expression is independent of KC differentiation [10] and this resulted in the induction of pro-inflammatory cytokine expression in both undifferentiated and differentiated KCs (Figure S1). In contrast to differentiated uninfected KCs, the hrHPV+ KCs that expressed TLR9 after differentiation, failed to induce the expression of *IFNβ, IL-8 and MIP3α* upon incubation with CpG (Figure 1C), indicating that PRR-signaling can be suppressed in undifferentiated and differentiated hrHPV+ KCs.

**Figure 2:**
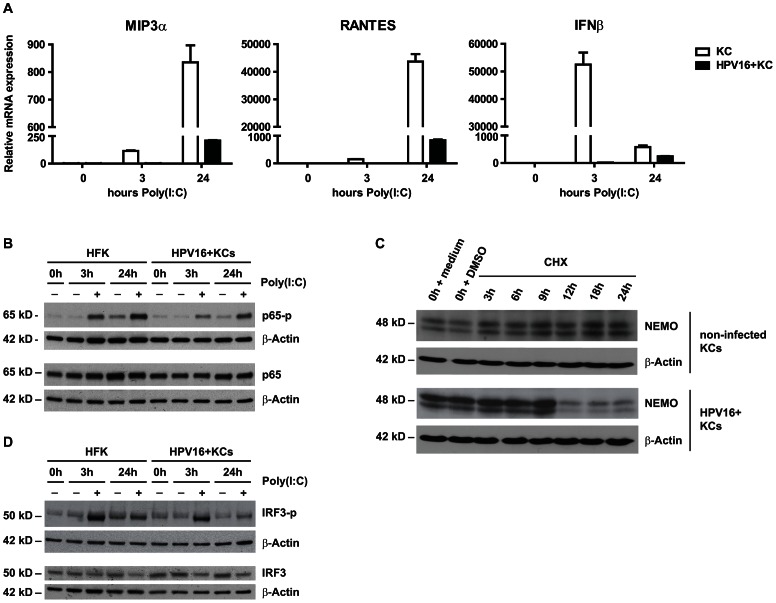
Canonical NF-κB signaling is impaired upstream of the transcription factor p65. (**A**) Poly(I∶C) induced cytokine expression in HPV16+ KCs compared to non-infected KCs. *MIP3a*, *RANTES* and *IFNβ* expression was measured by qRT-PCR. Gene expression was normalized using *GAPDH* mRNA levels and standardized against 0 h of stimulation with poly(I∶C). (**B**) Poly(I∶C) stimulated phosphorylation levels of p65 in HPV16+ KCs compared to non-infected KCs. Total p65 levels and p65 phosphorylation status were determined in whole cell extracts by western blotting. β-actin served as loading control. (**C**) NEMO degradation in HPV16+ KCs compared to non-infected KCs. Monolayer cultures were treated with 100 µM cycloheximide (CHX) and harvested after 0, 3, 6, 9, 12, 18 and 24 hours. Whole cell extracts were analyzed by western blotting using antibodies against NEMO and β-actin (control for protein degradation). (**D**) Poly(I∶C) stimulation-induced phosphorylation levels of IRF3 in hrHPV+ KCs compared to KCs. Total IRF3 levels and IRF3 phosphorylation status were determined in whole cell extracts by western blotting. β-actin served as loading control.

As the basal KCs are the target for hrHPV and TLR9 is not functionally expressed in basal KCs and hrHPV+ KCs displayed an impaired production of cytokines in response to poly(I∶C), subsequent studies were performed in the context of poly(I∶C) stimulation. In addition to the secretion of cytokines, also the gene expression levels of *MIP3α*, *CCL5* (*RANTES*) and *IFNβ* in hrHPV+ KCs were lower when compared to uninfected KCs upon 3 or 24 hours of poly(I∶C) stimulation (Figure 2A).

The production of pro-inflammatory cytokines and chemokines upon activation of the NF-κB pathway requires the phosphorylation and nuclear translocation of the subunit p65 [6]. The levels of phosphorylated p65 were lower in poly(I∶C) stimulated hrHPV+ KCs than in non-infected KCs (Figure 2B), suggesting that the functional impairment of PRR signaling occurs upstream of this molecule. The IKK complex is a key component of the poly(I∶C)-induced NF-κB pathway, with NEMO (IKKγ) functioning as a scaffold. The degradation of NEMO may form a mechanism for viruses to avoid innate immune signaling [16], [17]. Therefore, the effect of hrHPV on the protein levels of NEMO was analyzed. Following treatment of non-infected KCs and hrHPV+ KCs with cycloheximide (CHX) – to prevent new protein synthesis – it became clear that NEMO degradation was enhanced in hrHPV+ KCs (Figure 2C and Figure S2), thereby explaining the decreased phosphorylation of p65 observed.

The production of type I IFN (*e.g.* IFNβ) requires the activation of cytosolic IRF3 by phosphorylation and subsequent translocation to the nucleus. Analysis of poly(I∶C) stimulated KCs and hrHPV+ KCs suggested that also the levels of phosphorylated IFR3 levels were decreased in HPV+ KCs (Figure 2D).

### The high risk HPV viral transcript is needed to impair PRR signaling

**Figure 3:**
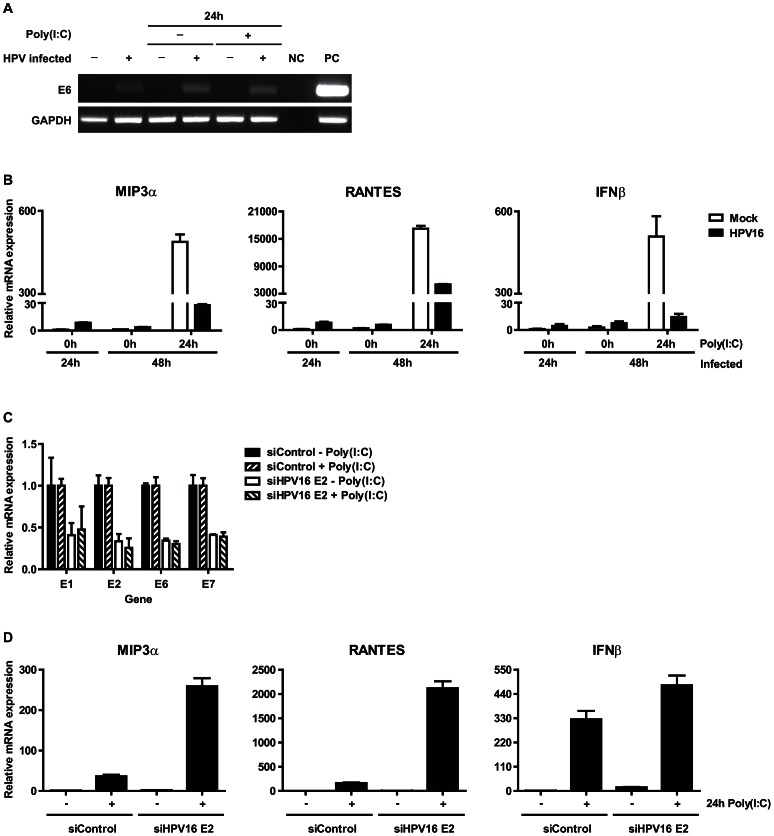
Expression of human papillomaviral transcripts are required to impair cytokine expression of poly(I∶C) stimulated keratinocytes. (**A**, **B**) Cytokine expression at the initial stage of HPV16 infection. Primary basal layer human foreskin keratinocytes were infected with native HPV16. (**A**) Viral early gene E6 expression was analyzed 1 and 2 (24 h poly(I∶C)) days after infection by PCR. NC: negative control, PC: positive control, HPV16+ KCs. (**B**) *MIP3a*, *RANTES* and *IFNβ* expression was measured by qRT-PCR. Gene expression was normalized against *GAPDH* mRNA levels and standardized against the 0 h poly(I∶C) stimulated non-infected cells. Similar results were observed in two independent experiments. (**C**, **D**) Poly(I∶C)-induced cytokine expression in HPV+ KCs transfected with control siRNA (siControl) or siRNA targeting HPV16 E2 (siHPV16 E2). *E1*, *E2*, *E6*, *E7* (**C**) as well as *MIP3a*, *RANTES*, and *IFNβ* (**D**) expression was analyzed by qRT-PCR. Gene expression was normalized against *GAPDH* mRNA levels and standardized against no poly(I∶C) siControl. For all three genes the response to poly(I∶C) was significantly higher when HPV16 E2 was suppressed (p<0.001, one-way ANOVA).

To confirm that the impairment in the production of IFNβ and pro-inflammatory cytokines did not simply reflect biological differences between the different primary KCs used but indeed was caused by hrHPV, we infected primary keratinocytes with infectious HPV16 virions (Figure 3A) for 24 hours and then stimulated the non-infected and newly infected KCs with poly(I∶C) for another 24 hours after which the levels of *IFNβ*, *RANTES* and *MIP3α* transcripts were measured (Figure 3B). After 24 hours of infection there was a small but discernible increase in the levels of these genes indicating that the keratinocytes initially react to the presence of the virus. However, the levels already dropped at 48 hours post-infection indicating that the virus rapidly exerted its PRR-signaling inhibitory effects. In addition, at the same time point these newly hrHPV-infected keratinocytes displayed a hampered activation of *IFNβ*, *RANTES* and *MIP3α* following 24 hours of stimulation with poly(I∶C) (Figure 3B). Moreover, we repressed the polycistronic viral mRNA transcript [18], [19] in hrHPV+ KCs by the use of siRNA targeting HPV16 *E2* as this allows the destruction of the whole RNA chain. Indeed the suppression of HPV early gene E2 expression translated into an overall decrease in viral early gene expression (Figure 3C) and an increase in the transcription of *IFNβ*, *RANTES* and *MIP3α* following poly(I∶C) stimulation (Figure 3D).

Together these data demonstrate that the innate immune response to viral and bacterial-derived PRR stimuli of both undifferentiated and differentiated hrHPV+ keratinocytes is suppressed by HPV at a point downstream of the PRR receptors but upstream of the transcription factors that relay the PRR signals to the nucleus.

### The ubiquitin-modifying enzyme UCHL1 is over-expressed in hrHPV-positive keratinocytes and responsible for suppressing the production of type I IFN as well as pro-inflammatory and chemotactic cytokines

**Figure 4:**
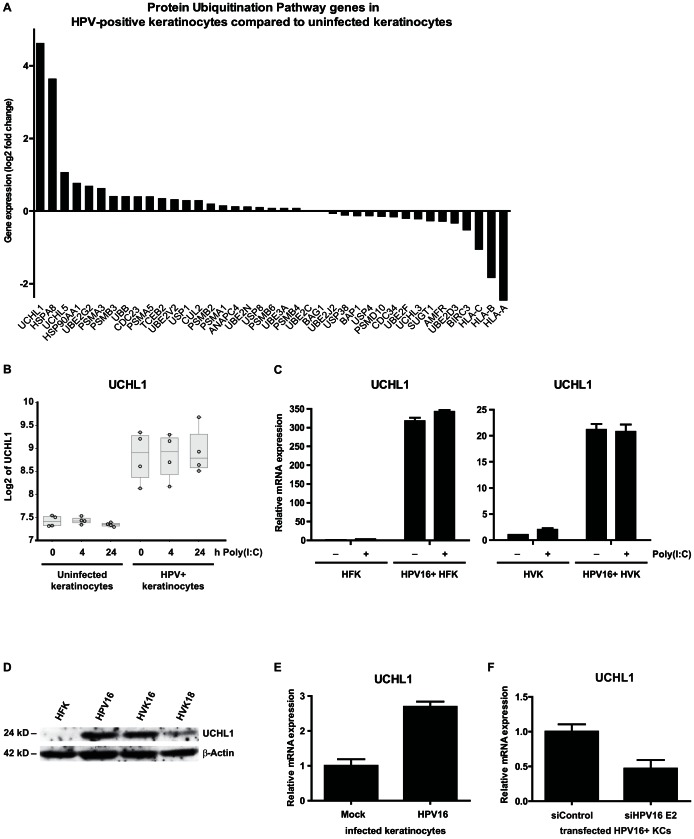
HPV induces expression of *UCHL1* in keratinocytes. (**A**) Summary of all differentially expressed genes within the Protein Ubiquitination Pathway. Differentially expressed genes between four uninfected KC and four hrHPV+ KC cultures with adjusted *p*-value≤0.05 identified 24 hours after poly(I∶C) stimulation by microarray analysis (log2 ratios) are shown. (**B**) *UCHL1* microarray gene expression values (log2 intensities) after 0, 4, and 24 hours of poly(I∶C) stimulation in four primary KCs and four hrHPV+ KCs (circles). The box represents the 25^th^ and 75^th^ percentiles, the median is indicated with a horizontal line within the box, and the whiskers represent the minimum and maximum. (**C**) *UCHL1* expression in HPV16+ human foreskin keratinocytes (HFK; left panel) and HPV16+ human vaginal keratinocytes (HVK; right panel) when compared to uninfected KCs. KCs were either left unstimulated or stimulated with poly(I∶C) for 24 hrs. *UCHL1* expression was normalized against *GAPDH*. (**D**) UCHL1 protein levels in HPV16+ human foreskin keratinocytes (HPV16) and HPV16+ or HPV18+ human vaginal keratinocytes (HVK16 or HVK18, respectively) when compared to non-infected KCs (HFK) as detected by western blotting (WB) in whole cell extracts. β-actin served as loading control. (**E**) *UCHL1* expression at the initial stage of HPV16 infection. Primary basal layer human foreskin keratinocytes were infected with native HPV16 (HPV16 infected keratinocytes) or not (Mock). *UCHL1* mRNA expression was analyzed by qRT-PCR 2 days after infection. Gene expression was normalized against *GAPDH* mRNA levels and standardized against the non-infected cells. Similar results were observed in two independent experiments. (**F**) *UCHL1* expression in HPV+ KCs transfected with control siRNA (siControl) or siRNA targeting HPV16 E2 (siHPV16 E2). *UCHL1* expression was analyzed by qRT-PCR. Gene expression was normalized against *GAPDH* mRNA levels and standardized against siControl. Similar results were observed in more than 3 independent experiments.

Our data suggest that hrHPV+ keratinocytes manifest a generalized inability to respond to stimulation through interference at, or downstream of the cytosolic part of the PRR signaling pathways. We therefore re-analyzed the genome-wide expression profiles (Gene Expression Omnibus accession number GSE21260) of several different uninfected KC cultures and hrHPV+ KC cultures reported previously [10] by Ingenuity Pathways Analysis (IPA) and found a highly significant enrichment of genes belonging to the protein ubiquitination pathway (Table S1; p = 6.69×10^−5^). In this pathway, the gene for the enzyme ubiquitin carboxyl-terminal hydrolase L1 (*UCHL1*) was the most upregulated gene in hrHPV+ KCs compared to uninfected KCs (Figure 4A and B). The upregulation of *UCHL1* in hrHPV+ KCs was confirmed by RT-qPCR in both foreskin and vaginal epithelial hrHPV+ KC cultures and expression was not influenced by poly(I∶C) activation (Figure 4C). Furthermore, UCHL1 upregulation at the protein level was tested and shown for three different hrHPV+ KCs by western blotting (Figure 4D). Moreover, expression of *UCHL1* was upregulated 2 days post-infection of HPV16 in primary keratinocytes when compared to mock-infected primary keratinocytes (Figure 4E), whereas knock-down of the polycistronic viral mRNA transcript in hrHPV+ KCs by siRNA for HPV16 E2 resulted in a decreased *UCHL1* expression (Figure 4F). Thus, the cellular deubiquitinase UCHL1 is upregulated by hrHPV.

**Figure 5:**
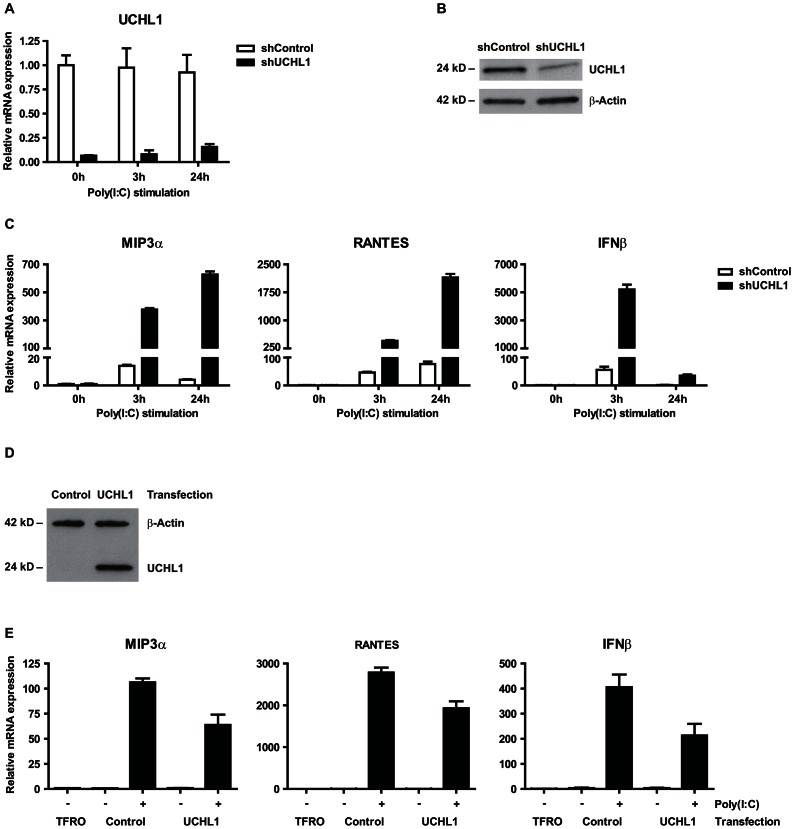
*UCHL1* is responsible for suppressing poly(I∶C) mediated gene activation of IFN-I and proinflammatory cytokines in hrHPV-infected KC. (**A**–**C**) *UCHL1* knock-down effect of poly(I∶C) mediated gene expression of IFN-I and proinflammatory cytokines. HPV16+ keratinocytes were transduced with lentiviral vectors expressing shRNA against control mRNA (TurboGFP; shControl) or targeting mRNA of UCHL1 (shUCHL1). Cells were either left unstimulated, or were stimulated with poly(I∶C) for 3 or 24 hrs. (**A**) *UCHL1* mRNA expression was analyzed by qRT-PCR and (**B**) UCHL1 protein levels were analyzed by western blotting in whole cell extracts, β-actin served as loading control. (**C**) *MIP3α*, *RANTES* and *IFNβ* mRNA expression was analyzed by qRT-PCR. Gene expression was normalized against *GAPDH* mRNA levels and standardized against 0 h of stimulation with poly(I∶C). (**D**, **E**) *UCHL1* overexpression effect on the activation of poly(I∶C) mediated gene expression of *IFNβ* and proinflammatory cytokines. Uninfected keratinocytes were transfected with a vector harboring the *UCHL1* gene, an empty control or only received the transfection agent (TFRO). Cells were either left unstimulated, or were stimulated with poly(I∶C) for 24 hrs. (**D**) UCHL1 protein levels were upregulated in the *UCHL1*-transfected cells as detected by western blotting in whole cell extracts, β-actin served as loading control. (**E**) *MIP3α* and *RANTES* mRNA expression was analyzed by qRT-PCR. Gene expression was normalized against *GAPDH* mRNA levels and standardized against the TFRO at 0 h of stimulation with poly(I∶C).

Although UCHL1 had not been associated with the inhibition of PRR signaling, its enhanced expression in hrHPV+ KCs fits well with the general role of deubiquitinases in controlling PRR signaling [6]. To test whether hrHPV-induced UCHL1 inhibits PRR signaling, we used lentiviral vectors expressing short-hairpin RNA (shRNA) against *UCHL1* and this resulted in a downregulated expression of *UCHL1* transcripts and protein levels in hrHPV+ KCs (Figure 5A and B). Upon stimulation with poly(I∶C), hrHPV+ KCs expressing shRNA against *UCHL1* (shUCHL1) but not hrHPV+ KCs expressing a control shRNA (shControl) restored poly(I∶C)-mediated induction of type I interferon and proinflammatory cytokines (Figure 5C). Similar results were obtained using transiently transfected RNA interference (RNAi) oligos targeting *UCHL1* but not with control RNAi oligos (Figure S3). An increase in the expression levels of *IL8* and *MIP3α* was detected in hrHPV+ KCs in which *UCHL1* was downregulated. Gene expression increased to the same levels found in *UCHL1*-non silenced hrHPV+ KCs cells stimulated with poly(I∶C) (Figure S3). This suggests that downregulation of *UCHL1* increases the gene expression of *IL-8* and *MIP3α* in hrHPV+ KCs. Conversely, transfection of uninfected KCs to overexpress *UCHL1* resulted in a decreased expression of *MIP3α*, *RANTES* and *IFNβ* upon poly(I∶C) stimulation (Figure 5D and E). Based on control experiments in which KCs were transfected with green fluorescent protein expressing plasmids, the transfection efficiency of keratinocytes was 30–40% (not shown), indicating that in a large part of the keratinocytes the activation of cytokine-encoding genes is not impaired and explaining the expression levels of these cytokine-encoding genes that are still detected.

All together, these data clearly demonstrate that UCHL1 can downregulate the PRR-mediated activation of both the type I IFN and proinflammatory cytokine and chemokine pathways.

### Knock down of UCHL1 increases the phosphorylation of IRF3 and NFκB p65 and alleviates NEMO degradation

**Figure 6:**
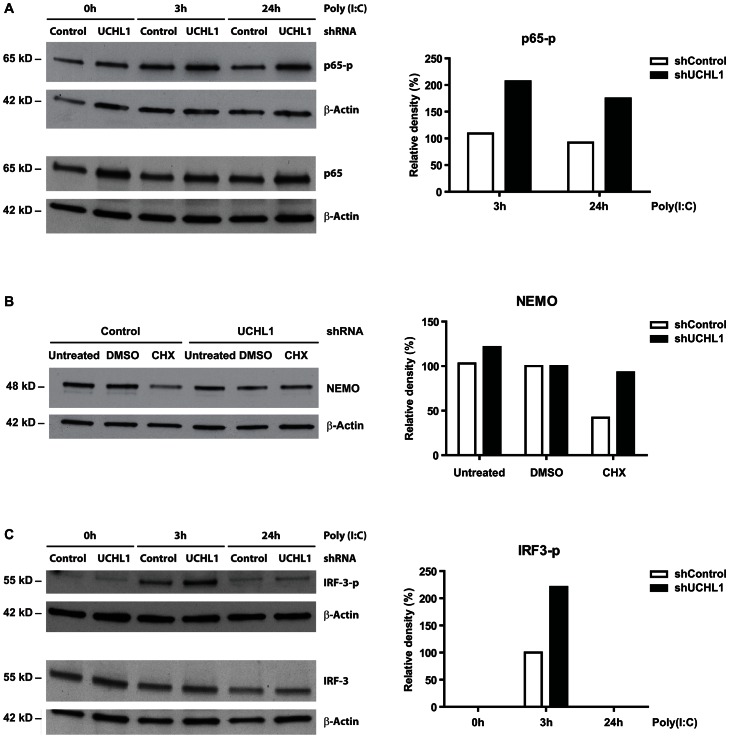
*UCHL1* reduces phosphorylation levels of IRF3 and p65 and degrades NEMO in hrHPV-positive KC. (**A**) *UCHL1* knock down effect on poly(I∶C) stimulated p65 phosphorylation in HPV16+ keratinocytes. Monolayer cultures of shControl or shUCHL1-expressing HPV16+ KCs were stimulated for 0, 3 or 24 hours with Poly(I∶C). Whole cell extracts were analyzed by western blotting for p65, p65-p and β-Actin (as loading control). The relative expression of p65-p was quantified by measuring its density and by normalizing it to that of β-Actin. The expression levels of p65-p in the 0 h Poly(I∶C) cells were set to 100% for both shControl and shUCHL1 cells. The p65-p levels in the 3 h and 24 h Poly(I∶C) cells were calculated against the levels measured at 0 h Poly(I∶C) (right panel). (**B**) NEMO protein levels after knock down of *UCHL1* in HPV16+ KCs. Monolayer cultures of shControl or shUCHL1-expressing HPV16+ KCs were treated with 100 µM cycloheximide (CHX) for 16 hours. Whole cell extracts were analyzed by western blot using antibodies against NEMO and β-Actin (control for protein content). The relative expression of NEMO was quantified by measuring its density and by normalizing it to that of β-Actin. The expression of NEMO in the DMSO control was set to 100% (right panel). (**C**) *UCHL1* knock down effect on poly(I∶C) stimulated IRF3 phosphorylation in HPV16+ keratinocytes. Similar to A, however cell extracts were analyzed by western blotting using antibodies against IRF3, IRF3-p and β-Actin (as loading control). The relative expression of IRF3-p was quantified by measuring its density and by normalizing it to that of β-Actin. The expression of IRF3-p in the 3 h Poly(I∶C) control cells (no knock down of *UCHL1*) was set to 100% (right panel).

We then asked whether the restoration of PRR signaling, as indicated by an increased induction of type I interferon and proinflammatory cytokines by the knock down of UCHL1 in hrHPV+ KCs would also be reflected in the levels of phosphorylated p65 (p65-p) and IRF3 (IRF3-p) upon poly(I∶C) stimulation. Therefore, the p65-p and IRF3-p levels were analyzed in whole cell extracts of HPV16+ KCs stably expressing shRNA against *UCHL1* or control shRNA and following 3 h or 24 h of stimulation with poly(I∶C). Knock down of UCHL1 in hrHPV+ KCs resulted in increased p65 phosphorylation at 3 and 24 hours after poly(I∶C) stimulation (Figure 6A) coinciding with enhanced cyto- and chemokine production (Figure 5C). In addition, analysis of hrHPV+ KCs treated with cycloheximide revealed that NEMO degradation was alleviated when UCHL1 was knocked down by shUCHL1 as compared to the shControl hrHPV+ KCs (Figure 6B). Furthermore, higher levels of phosphorylated IRF3 were detected in hrHPV+ KCs in which *UCHL1* was knocked down as compared to hrHPV+ KCs expressing the shControl after 3 hours of poly(I∶C) stimulation (Figure 6C).

### UCHL1 alters the poly-ubiquitination of TRAF3 and NEMO

**Figure 7:**
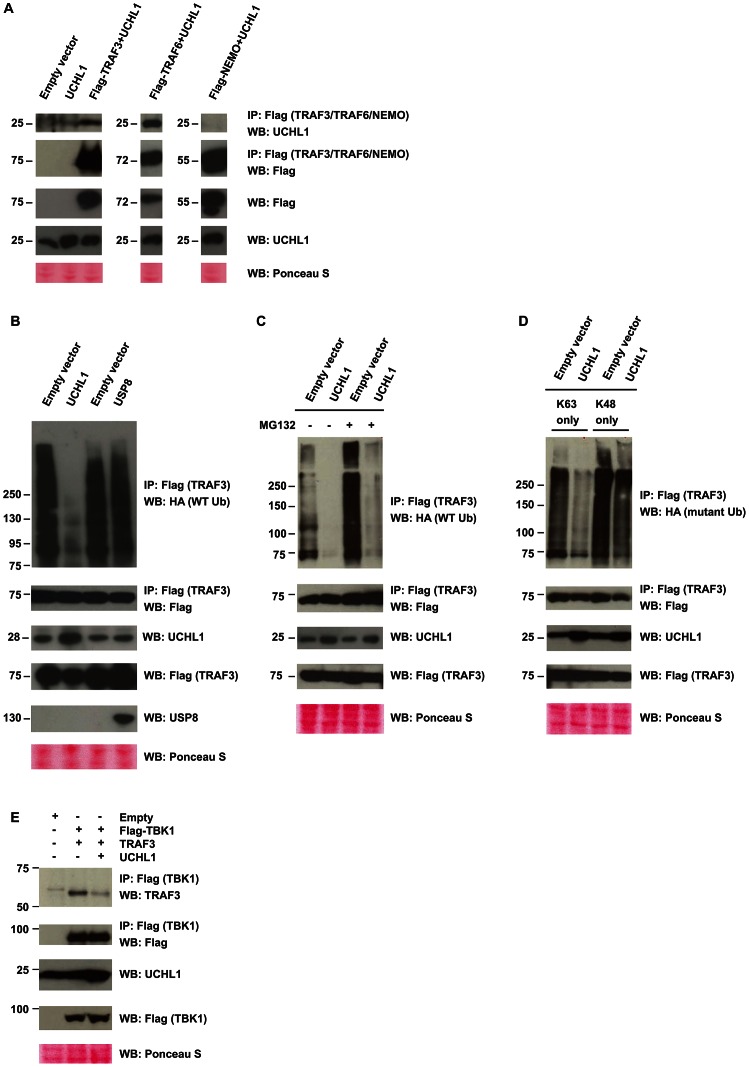
Interaction of UCHL1 with the PRR downstream signaling molecule TRAF3. (**A**) UCHL1 directly interacts with TRAF3 and TRAF6 but not NEMO. HEK293T cells were co-transfected as indicated and the respective TRAF3, TRAF6 or NEMO proteins were immunoprecipitated using Flag antibody, and co-precipitating UCHL1 was detected by WB. As a control a WB analysis for Flag was performed indicating that both TRAF3 and NEMO were present. The bottom three panels show a WB analysis of Flag and UCHL1 of non- immunoprecipitated lysate and a Ponceau S stained loading control for WB. (**B**) UCHL1, but not the control ubiquitin-specific protease 8 (USP8) mediates deubiquitination of TRAF3. HEK293T cells were co-transfected with Flag-TRAF3, HA-tagged wild-type ubiquitin (WT-Ub), and with either empty vector, WT UCHL1 or USP8. TRAF3 was immunoprecipitated with Flag antibody and WB was done with HA or Flag antibodies (top panels). The bottom four panels show a WB analysis of Flag, UCHL1, and USP8 of non- immunoprecipitated lysate and a Ponceau S stained loading control for WB. (**C**) Deubiquitination but not degradation of TRAF3 by UCHL1. HEK293T cells were co-transfected with Flag-TRAF3, HA-tagged wild-type ubiquitin (WT-Ub), and with either empty vector or WT UCHL1. Cells were left untreated or treated with proteasome blocker MG132. TRAF3 was immunoprecipitated with Flag antibody and WB was done with HA or Flag antibodies (top two panels). (**D**) UCHL1 mainly removes K63-linked poly-ubiquitin chains of TRAF3. HEK293T cells expressing Flag-TRAF3, HA-tagged mutant ubiquitin either K63 Only or K48 Only, and WT UCHL1 were immunoprecipitated with Flag antibody and analyzed by HA or Flag antibodies (top two panels). (**E**) UCHL1 lowers TRAF3-TBK1 complex formation. HEK293T cells were co-transfected and TBK1 was immunoprecipitated using Flag antibody, and co-precipitating TRAF3 or TBK1 was detected by WB (top two panels).

TRAF3 ubiquitination is critical for type I IFN production and is a likely target for ubiquitin-modifying enzymes such as UCHL1. As the biochemical experiments to understand the nature of this interaction would require substantial amounts of primary KCs, which can only grow for a few passages thereby restricting their use in biochemical studies, we switched to the HEK293T cell system that is widely used for these purposes. To investigate the interaction between UCHL1 and TRAF3 we overexpressed UCHL1 and Flag-tagged TRAF3 in HEK293T cells. After FLAG immunoprecipitation, we confirmed that UCHL1 co-immunoprecipitated with TRAF3 (Figure 7A). TRAFs are activated by oligomerization and auto-ubiquitination, a process that results in lysine 63 (K63)-linked poly-ubiquination of TRAF, and this event can be induced by either their overexpression or by receptor activation. In contrast K48-linked poly-ubiquitination results in proteasome-mediated degradation of ubiquitinated TRAFs [6]. To test whether UCHL1 modified TRAF3 ubiquitination status, Flag-tagged TRAF3 and haemagglutinin A (HA)-tagged ubiquitin were overexpressed in control or UCHL1 overexpressing HEK293T cells. Poly-ubiquitination of TRAF3 was clearly visible by immunoblot analysis but strongly reduced when UCHL1 was also overexpressed (Figure 7B, Figure S4). No reduction in poly-ubiquitination was detected when as a control the growth regulated ubiquitin-specific protease 8 (USP8), which similar to UCHL1 displays carboxyl-terminal hydrolase activity, was overexpressed (Figure 7B). The UCHL1-associated decreased detection of poly-ubiquitinated TRAF3 was not the result of increased TRAF3 degradation as blocking the proteasomal degradation pathway by the inhibitor MG132 did not result in a reappearance of poly-ubiquitinated TRAF3 (Figure 7C). Instead, experiments in which HA-tagged ubiquitin mutants ‘K63 Only’ and ‘K48 Only’ (where all lysine residues, except at position K63 and K48, respectively, were mutated to arginine) showed that UCHL1 removed K63-linked poly-ubiquitins but not K48-linked poly-ubiquitins (Figure 7D), consistent with the known deubiquitinating capacity of UCHL1 [20]. K63-linked ubiquitination is required for TRAF3 to bind its partner TBK1 to activate the downstream type I IFN-signaling pathway. As expected, UCHL1-mediated deubiquitination of TRAF3 resulted in less TRAF3 bound to TBK1 in UCHL1 overexpressing cells when compared to control cells (Figure 7E). These data clearly show that UCHL1 binds and deubiquitinates TRAF3 resulting in a decreased TRAF3-TBK1 complex formation.

**Figure 8:**
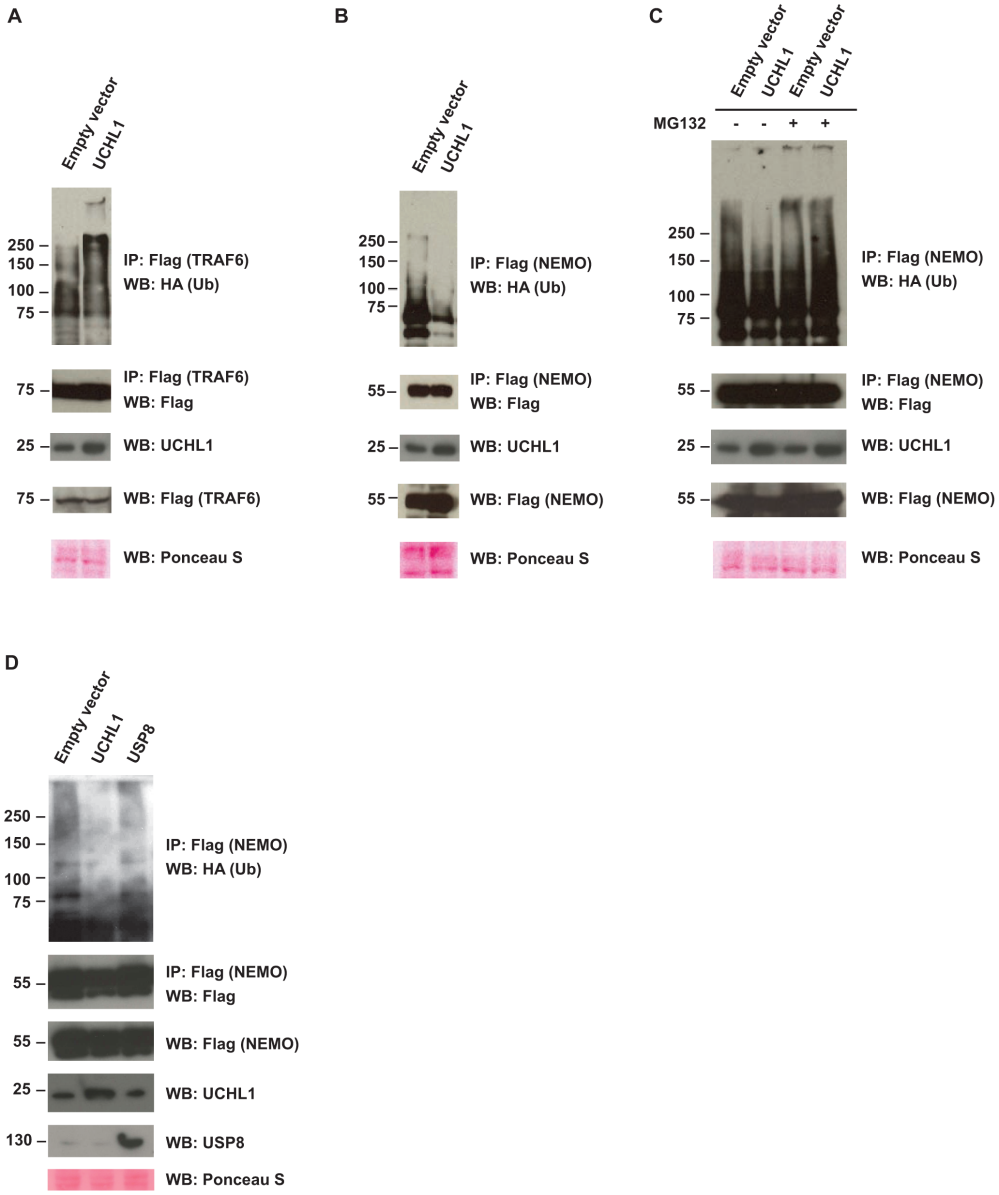
Interaction of UCHL1 with the PRR downstream signaling molecules TRAF6 and NEMO. (**A**) UCHL1 overexpression results in a modest poly-ubiquitination of TRAF6. HEK293T cells were co-transfected with Flag-TRAF6, HA-tagged WT-Ub, and with either empty vector or WT UCHL1. TRAF6 was immunoprecipitated with Flag antibody and western Blotting (WB) was done with HA or Flag antibodies (top two panels). The bottom three panels show a WB analysis of UCHL1 and Flag of non-immunoprecipitated lysate and a Ponceau S stained loading control for WB. (**B**) The effect of UCHL1 on NEMO. HEK293T cells were co-transfected with Flag-NEMO, HA-tagged WT-Ub, and with either empty vector or WT UCHL1. NEMO was immunoprecipitated with Flag antibody and WB was done with HA or Flag antibodies (top two panels). (**C**) The overexpression of UCHL1 mediates the degradation of NEMO. HEK293T cells were co-transfected with Flag-NEMO, HA-tagged WT-Ub, and with either empty vector or WT UCHL1. Cells were left untreated or were treated with MG132, NEMO was immunoprecipitated with Flag antibody and WB was done with HA or Flag antibodies (top two panels). (**D**) USP8 does not deubiquitinate NEMO. HEK293T cells were co-transfected with Flag-NEMO, HA-tagged wild-type ubiquitin (WT-Ub) and UCHL1 or USP8. NEMO was immunoprecipitated with Flag antibody and WB was done with HA antibodies (top panel). The bottom four panels show a WB analysis of Flag, UCHL1, and USP8 of non-immunoprecipitated lysate and a Ponceau S stained loading control for WB.

Poly-ubiquitination of TRAF6 and its downstream partner NEMO is critical for the PRR-induced activation of proinflammatory cytokine genes [6]. Since the overexpression of UCHL1 clearly affected proinflammatory cytokine synthesis (Figure 5) the interaction of UCHL1 with TRAF6 and NEMO was tested. Co-expression and immunoprecipitation experiments in HEK293T cells showed that UCHL1 bound to TRAF6 but not to NEMO (Figure 7A). In contrast to what we observed for TRAF3, UCHL1 displayed a modest effect on the poly-ubiquitination of TRAF6 (Figure 8A). However, poly-ubiquitination of NEMO was reduced in UCHL1 overexpressing cells (Figure 8B, Figure S4) but not in USP8 overexpressing cells (Figure 8D). Inhibition of proteasome function by MG132 suggested that the reduced poly-ubiquitination of NEMO was the result of enhanced degradation of NEMO in cells overexpressing UCHL1 (Figure 8C, compare lanes 2 and 4), albeit that the total protein levels of NEMO in these transfected cells remained unaffected. This is not unexpected as also in the endogenous setting (Figures 2 & 6) the degradation of NEMO could only be visualized when the hrHPV+ KCs where pretreated with cycloheximide to prevent new protein synthesis.

Collectively, these data support the notion that UCHL1 can suppress the PRR-signaling pathways necessary for type I IFN and pro-inflammatory cytokine production by the removal of the activating K63 ubiquitins from TRAF3 and the forced degradation of NEMO.

## Discussion

We have employed a unique model for hrHPV infection to examine the potential mechanisms underlying the capacity of hrHPV to evade host immunity by suppression of the innate immune response [10]. We utilized primary KC cultures that were newly infected with HPV16 virions or primary KCs stably maintaining the episomal hrHPV genome to show that despite the expression of multiple PRRs the production of IFNβ and pro-inflammatory cytokines and chemokines is suppressed by hrHPV as a consequence of reduced PRR signaling. We provided firm evidence that this suppression depends on the hrHPV-induced upregulation of the cellular ubiquitin-modifying enzyme UCHL1 in infected primary KCs.

Finally, classical biochemical studies in HEK293T cells [11], [21], [22] performed to understand how UCHL1 mechanistically could suppress the production of type I interferons and pro-inflammatory cytokines revealed that UCHL1 regulated the ubiquitination of the PRR-signaling pathway adaptor molecules TRAF3 and NEMO. UCHL1 removes activating K63-linked ubiquitin molecules from TRAF3 resulting in a lower amount of the downstream signaling complex TRAF3-TBK-1 to suppress the type I IFN pathway. This puts UCHL1 within the family of other deubiquinating enzymes that regulate the PRR pathways by selectively cleaving lysine-63 (K63)-linked ubiquitin chains from TRAFs (e.g. DUBA, OTUB1, OTUB2, A20) [21], [22], [23], [24], [25], [26]. Furthermore, we showed that UCHL1 bound to TRAF6 and mediated the enhanced degradation of NEMO as a mechanism to suppress the proinflammatory cytokine NF-κB pathway. Notably, the ubiquitin-modifying enzyme A20, a known negative regulator of the TLR pathway, has two ubiquitin-editing domains allowing it to remove and to add ubiquitin chains (22, 26). UCHL1 has also been reported to have these two opposing functions (20). The ligase activity of UCHL1 may explain the ubiquitination of TRAF6 observed in our study. Although UCHL1 did not bind to NEMO, it is known that other deubiquitinating enzymes (*e.g.* CYLD, A20) bind to TRAFs in order to dock on the IKK complex and to associate with NEMO [21], [27]. TRAF6-dependent poly-ubiquitination of NEMO is well known [28]. It is highly likely that UCH-L1 acts in a similar fashion and this would fit with TRAF6-NEMO interaction and our observations that NEMO is degraded.

Our data on the suppression of NF-κB signaling via the degradation of NEMO by UCHL1 fits well with earlier observations concerning the overexpression of UCHL1 in vascular cells. Here UCHL1 attenuated TNF-α induced NF-κB signaling and this was associated with stabilization of IκBα and a decrease in its basal ubiquitination [29]. The activation of NF-κB signaling requires IκBα to become degraded following an interaction with the IκB kinase complex (IKK) which comprises NEMO. Hence, the degradation of NEMO may explain previous observations on UCHL1-associated stabilization of IκBα.

UCHL1 is not found to be central in the network of genes affected by hrHPV, suggesting that it is not part of the cellular genes affected in order to assist in HPV genome replication and viral protein production [10]. This indicates that UCHL1 is not directly involved in viral propagation but rather recruited by hrHPV to suppress keratinocyte-mediated production of cytokines and chemokines that would result in the attraction and activation of an adaptive immune response, thereby enabling the virus to persist and propagate.

Many viruses utilize multifunctional viral proteins in order to evade NF-κB- and IRF-mediated immune responses, to favor viral replication and/or to modulate cellular apoptosis and growth pathways [30]. The group of pox viruses have evolved to inhibit NF-κB-signaling by targeting one or more of the many different molecules of this signaling cascade [31]. The vaccinia virus B14 protein is known to inhibit NF-κB signaling by a variety of toll-like receptor agonists at the level of the IKK complex, of which NEMO is a member [32]. The vaccinia virus A64R protein inhibits TRIF-TRAF3-IRF signaling [33]. The pathogenic NY-1 hantavirus Gn protein inhibits TRAF3 signaling by blocking the formation of TBK1-TRAF3 complexes [34] whereas the LMP1 protein of Epstein-Barr virus directly binds to TRAF3 [35]. Furthermore, foot-and-mouth disease virus 3c protease cleaves NEMO [16] and cytomegalovirus M54 protein induces the proteasome-independent degradation of NEMO [17]. In contrast, human papillomaviruses, with a rather limited coding capacity in their genomes, rely for many aspects of their life cycle on the utilization of cellular proteins [36] and this includes the recruitment of different cellular E3 ligases to mediate degradation of cellular proteins through the ubiquitin-proteasomal pathway [37]. UCHL1 is one of the most abundant proteins in the mammalian nervous system and is involved in regulating synaptic transmission at the neuromuscular junctions [38]. Aberrant expression is related to Parkinson's disease [20] and is also implicated in oncogenesis [39]. In hrHPV+ keratinocytes UCHL1 is expressed and redirected to adopt a new function that is to serve as a negative regulator of the PRR-signaling pathway. As such it mimics the ubiquitin-modifying enzyme A20 which is the natural negative regulator of the TLR pathway [22], [26], [40]. UCHL1 interferes with the adaptor molecules TRAF3, TRAF6 and NEMO which all function at junctions for the immune stimulating signals from different PRR and type I IFNR to activate NF-κB- and IRF-mediated immune responses. Therefore, the utilization of UCHL1 represents a truly effective use of a cellular protein as it may suppress the immunostimulatory signals initiated through recognition of HPV genomic DNA by TLR9 [5] and RIG-I [11], [12] as well as those obtained via the cell surface receptors for type I IFN [41].

The high expression of UCHL1 in primary keratinocytes carrying infectious hrHPV [13], [14] is generally lost after transformation of these keratinocytes to tumor cells. Although transformed keratinocytes expressing un-physiologically high levels of *E6* and *E7* via retroviral transduction still may express *UCHL1*, only a minority of spontaneously HPV-transformed cervical carcinoma's and none of the well known HPV-induced cancer cell lines overexpress UCHL1 [42], indicating that under normal conditions *UCHL1* overexpression in HPV transformed cells is not a common event. The expression of the hrHPV oncoproteins E6 and E7 is required to maintain the transformed state of keratinocytes [2], [43] suggesting that it is not E6 or E7, but one or more of the other viral proteins responsible for upregulation of UCHL1 (currently under investigation). Previous studies on the innate immune response to hrHPV relied on the overexpression of hrHPV E6 and/or E7 proteins, showing that the viral DNA-sensing TLR9 was altered [8] and that overexpressed HPV E6 or E7 could bind to IRF3 [44] and/or the co-activator CPB [45]. Furthermore, overexpressed hrHPV E6 and/or E7 attenuated IκB kinase signaling [46], and interfered with the nuclear translocation of the interferon-stimulated gene factor 3 (ISGF3) transcription complex [47]. The fact that these studies were performed with only HPV E6- and E7 transfected or transformed cells may explain why the central role of UCHL1 in dampening immunity towards hrHPV+ keratinocytes was not discovered before. In addition, the loss of UCHL1 mediated suppression of the NF-κB pathway in hrHPV E6/E7-induced cancer cells fits well with the notion that solid tumors require the NF-κB-mediated expression of proteins that promote survival, proliferation, invasion and metastasis [48] which is acquired through the E6-mediated deactivation of CYLD [49], a negative regulator of TRAF2 and TRAF6-mediated activation of NF-κB [21], [24].

All together, our data implicate UCHL1 as a negative regulator of the PRR pathways helping hrHPV to evade host immunity and allowing it to persist in keratinocytes.

## Methods

### Cell culture

Primary cultures of human epithelial keratinocytes were established from foreskin [50] and vaginal tissues and grown in serum-free medium (Defined KSFM, Invitrogen, Breda, The Netherlands). Keratinocyte lines stably maintaining the full episomal HPV genome following electroporation were grown in monolayer culture using E medium in the presence of mitomycin C treated J2 3T3 feeder cells [13], [14] for two passages and were then adapted to Defined K-SFM for one passage before experimentation. None of the cell cultures were used after passage 15 and the non-transformed state of the cells used was confirmed by the expression of both *E1* and *E2* so that the cells used truly represent the preneoplastic state in which the HPV genomes remained episomal and were capable of the complete viral life cycle. Keratinocytes were terminally differentiated by placing them into serum-free medium containing 1.75% methyl cellulose and 1.8 mM Ca^2+^ for 24 hours [50]. Cells were harvested by washing out the methyl cellulose three times. HEK293T cells were cultured in Dulbecco's modified Eagle's medium supplemented with 10% fetal bovine serum, 2 mM l-glutamine and 1% penicillin-streptomycin (Gibco-BRL, Invitrogen). Transient transfections were performed using calcium phosphate or Lipofectamine 2000 (Invitrogen).

### HPV16 infection of non-infected keratinocytes

Primary basal layer human foreskin keratinocytes were seeded at 7.5×10^4^ cells per well of a 24-wells plate in K-SFM and then allowed to attach for 48 hours. Cells received fresh medium (Mock infected) or medium containing native HPV16 isolated from raft cultures at a MOI 100 for 24 hours. Cells were stimulated with or without 25 ug/ml poly(I∶C) in K-SFM for 0 or 24 hours and harvested at the indicated time-points.

### Plasmid construction

Full length human cDNA clones for UCHL1, TRAF3, TRAF6 and TBK1 were obtained from Open Biosystems (Surrey, UK). The cDNA clones were PCR amplified and subcloned either into pcDNA3.1 expression vector or into Flag-tagged pcDNA3.1 vector. Full-length Flag-NEMO construct was kindly provided by Dr. C. Sasakawa, University of Tokyo, Japan [51]. HA-tagged wild-type and mutant ubiquitin constructs were kindly provided by Dr. A. Iavarone, Columbia University, USA.

### RNA expression analyses

Total RNA was isolated using TRIzol (Invitrogen) according to manufacturer's instructions. RNA was purified using RNeasy Mini Protocol (Qiagen, Venlo, The Netherlands). Total RNA (0.2 µg) was reverse transcribed using SuperScript III reverse transcriptase (Invitrogen) and oligo dT primers (Promega, Madison, USA). TaqMan PCR was performed using TaqMan Universal PCR Master Mix and pre-designed, pre-optimized primers and probe mix for IL-8, MIP-1α, MIP-3α, RANTES, IL-1β, IFNβ, UCHL1 and GAPDH (Applied Biosystems, Foster City, USA). Threshold cycle numbers (Ct) were determined using the 7900HT Fast Real-Time PCR System (Applied Biosystems) and the relative quantities of mRNA per sample were calculated using the ΔΔCt method as described by the manufacturer using GAPDH as the calibrator gene.

### Stimulation of cells with TLR ligands and ELISA

5×10^5^ cells were plated in 1 ml in each well of 24-well flat bottom plate. Cells were left unstimulated or stimulated with Pam3CSK4 (5 µg/ml), Poly(I∶C) (25 µg/ml), LPS (3.33 µg/ml ), flagellin (150 ng/ml), R848 (1 µg/ml), CpG (1 µM) or TNFα (50 ng/ml) for 24 hours. Flagellin was a kind gift from Jean-Claude Sirard (Institut Pasteur, Lille, France). TLR ligands were purchased from Invivogen (San Diego, USA). The supernatants were harvested and IL-8, MIP-3α, and MIP-1α concentrations were determined using corresponding Quantikine ELISA kits (R&D Systems, Oxon, UK).

### RNAi and shRNA

Non-targeting RNAi oligos (ON-TARGET*plus* Non-targeting Pool, catalogue D-001810-10-20) and oligos targeting UCHL1 (ON-TARGETplus SMARTpool, catalogue L-004309-00) were purchased from Dharmacon (Chicago, IL). Cells were transfected with RNAi using N-TER Nanoparticle siRNA Transfection System (Sigma-Aldrich, St. Louis, MO) according to manufacturer's instructions. 24 hours after transfection, cells were stimulated with poly(I∶C) (25 µg/ml) for another 24 hours and experiments were performed.

The shRNA's used were obtained from the MISSION TRC-library of Sigma-Aldrich (Zwijndrecht, The Netherlands). The MISSION shRNA clones are sequence-verified shRNA lentiviral plasmids (pLKO.1-puro) provided as frozen bacterial glycerol stocks (Luria Broth, carbenicillin at 100 µg/ml and 10% glycerol) in Escherichia coli for propagation and downstream purification of the shRNA clones. pLKO.1 contains the puromycin selection marker for transient or stable transfection. The construct against UCHL1 (NM_004181) was TRCN0000011079 (LV079): CCGGCAGTTCTGAAACAGTTTCTTTCTCGAGAAAGAAACTGTTTCAGAACTGTTTTT and the control was: SHC004 (MISSION TRC2-pLKO puro TurboGFP shRNA Control vector): CCGGCGTGATCTTCACCGACAAGATCTCGAGATCTT GTCGGTGAAGATCACGTTTTT. HPV16+ KCs were seeded 7.5×10^4^ cells per well to a 12-wells plate in K-SFM and were allowed to attach over night. Medium was replaced by infection medium (K-SFM+30% virus supernatant; MOI = 5), containing either the lentivirus LV079 in IMDM 5% FCS or as control SHC004. HPV16+ KCs were infected over night after which infection medium was replaced by K-SFM containing 1000 ng/ml puromycin for 48 hours to select for successfully infected HPV16+ KCs. Then the medium was replaced by K-SFM without puromycin and cells were grown for 24 hours. To stimulate the PRR pathways lentivirus-infected HPV16+ KCs were given K-SFM containing either no poly(I∶C) (two wells) or 25 ug/ml poly(I∶C) and were cultured for 21 hours. Then one of the two non-stimulated wells received 25 ug/ml poly(I∶C) and all cells were cultured for another 3 hours. Cells were harvested and total RNA was isolated.

Silencer Select siRNA against *HPV16 E2* (AACACUACACCCAUAGUACAUtt) was designed using siRNA Target Finder software (Ambion, Invitrogen). Blast search revealed that the designed E2 siRNA does not match with the known human transcriptome. E2 and Negative control #2 (NC2) siRNA (sequence not provided by manufacturer) were purchased from Ambion. HPV16+ KCs were transfected with 50 nM siRNA E2 or NC2 using Lipofectamine 2000 (Invitrogen) according to the manufacturer's instructions. 48 hours post-transfection cells received K-SFM containing no Poly(I∶C) or 25 ug/ml Poly(I∶C) and were cultured for 24 hours after which target gene expression was assayed by qRT-PCR.

### Western blot analysis and immunoprecipitation

For Western blotting, polypeptides were resolved by SDS–polyacrylamide gel electrophoresis (SDS–PAGE) and transferred to a PVDF membrane (Bio-Rad, Veenendaal, The Netherlands). Immunodetection was achieved with anti-Flag (1∶2000, Sigma-Aldrich), anti-HA (1∶1000, Covance), anti-TRAF3, anti-TRAF6 (both 1∶500, Santa Cruz, CA), anti-ubiquitin lysine 48-specific (1∶1000, Millipore, Amsterdam, The Netherlands), anti-poly-ubiquitin lysine 63 specific (1∶1000, Millipore), anti-TBK1 (1∶400, Santa Cruz), anti-NEMO (FL-419, Santa Cruz), anti-UCHL1 (1∶1000 Millipore, 1∶100 Abcam or 1∶1000 Santa Cruz), anti-USP8 (#8728, Cell Signaling Technology, Danvers, MA, USA), anti-phospho-p65 (Ser538; 1∶1000, #3033 Cell Signaling Technology) and anti-phospho-IRF3 (Ser396; 1∶2000, #4947, Cell Signaling Technology) or β-actin (1∶10,000, Sigma-Aldrich) antibodies. The proteins were visualized by a chemoluminescence reagent (Thermo Scientific, Etten-Leur, The Netherlands). X-Ray films were scanned using a GS-800 calibrated densitometer and Quantity One software (Bio-Rad, Veenendaal, The Netherlands) to quantify the intensity of the bands as a measure of the amount of protein of interest in the blot. The relative amount was determined by calculating the ratio of each protein over that of the density measured for the household protein β-Actin.

For immunoprecipitation, cells were collected after 48 h and then lysed in NP40 buffer supplemented with a complete protease inhibitor cocktail (Roche, Almere, The Netherlands). After pre-clearing with protein A/G agarose beads for 1 h at 4°C, whole-cell lysates were used for immunoprecipitation with either mouse or rabbit anti-Flag antibodies (Sigma-Aldrich), or rabbit anti-TRAF3 or rabbit anti-TRAF6. One to two µg of the antibody was added to 1 ml of cell lysate, which was incubated at 4°C for 2–3 h. After addition of protein A/G agarose beads, the incubation was continued for 1 h. Immunoprecipitates were extensively washed with lysis buffer and eluted with SDS loading buffer and boiled for 5 min. For immunoprecipitation under denaturing conditions, proteins were extracted using regular immunoprecipitation buffer plus 1% SDS and heated at 95°C for 5 min. The samples were diluted (10-fold) in regular immunoprecipitation buffer before immunoprecipitation.

## Supporting Information

Figure S1 **Cytokine production by poly(I∶C)-stimulated terminally differentiated keratinocytes.** *IL-8* and *MIP3α* expression levels in unstimulated or poly(I∶C)-stimulated uninfected KCs as examined by real-time PCR. KC were either left undifferentiated (undif) or terminally differentiated (terminal dif) with methylcellulose containing Ca^2+^. Gene expression was normalized using *GAPDH*.
(EPS)

Figure S2 **NEMO degradation depends on the expression of UCHL1.** NEMO degradation is enhanced in HPV16+ KCs but not in non-infected KCs. Monolayer cultures were treated with different concentrations of cycloheximide (CHX) for 24 hours. Whole cell extracts were analyzed by WB using antibodies against NEMO and β-actin (control for protein content).
(EPS)

Figure S3 **Restored cytokine production after knock down of** ***UCHL1*** **by RNAi oligos.** HPV16+ keratinocytes were transfected with non-targeting RNAi oligos and oligos targeting *UCHL1*. Cells were either left unstimulated, or were stimulated with poly(I∶C) for 24 hrs. *IL-8*,and *MIP3α* mRNA expression was analyzed by qRT-PCR. Gene expression was normalized against *GAPDH* mRNA levels.
(EPS)

Figure S4 **TRAF3 and NEMO are deubiquitinated by UCHL1.** HEK293T cells were co-transfected with HA-tagged wild-type ubiquitin (WT-Ub) only, with Flag-TRAF3 and HA-tagged wild-type ubiquitin (WT-Ub), and with Flag-TRAF3 and HA-tagged wild-type ubiquitin (WT-Ub) and UCHL1. A similar experiment was performed in which Flag-TRAF3 was replaced by Flag-NEMO (top panels). The bottom four panels show a WB analysis of Flag,Wt-Ub, and UCHL1 of non- immunoprecipitated lysate and a Ponceau S stained loading control for WB.
(EPS)

Table S1 **Enrichment of pathways between HPV-positive and uninfected keratinocytes as analyzed by Ingenuity Pathway Analysis (IPA).**
(DOC)
