# Supplementary material for: Human Papillomavirus (HPV) Upregulates the Cellular Deubiquitinase UCHL1 to Suppress the Keratinocyte's Innate Immune Response
Source: PLoS Pathog. 2013 May 23;9(5):e1003384. doi: 10.1371/journal.ppat.1003384 (PMC3662672; doi:10.1371/journal.ppat.1003384)
Supplement: Table S1 — Enrichment of pathways between HPV-positive and uninfected keratinocytes as analyzed by Ingenuity Pathway Analysis (IPA). (DOC) [file ppat.1003384.s005.doc]

**Enrichment of pathways between HPV-positive and uninfected keratinocytes as analyzed by Ingenuity Pathway Analysis (IPA).**

| **Canonical pathway** | ***p*-value** |
| --- | --- |
| Purine Metabolism | 1.15 x 10^-5^ |
| Oxidative Phosphorylation | 6.26 x 10^-5^ |
| **Protein Ubiquitination Pathway** | **6.69 x 10^-5^** |
| Graft-versus-Host Disease Signaling | 5.35 x 10^-4^ |
| LXR/RXR Activation | 7.55 x 10^-4^ |
| Mitochondrial Dysfunction | 8.22 x 10^-4^ |
| Nucleotide Excision Repair Pathway | 1.56 x 10^-3^ |
| Pyrimidine Metabolism | 1.15 x 10^-3^ |
| NRF2-mediated Oxidative Stress Response | 1.15 x 10^-3^ |
| Urea Cycle and Metabolism of Amino Groups | 1.15 x 10^-3^ |
| Inositol Metabolism | 1.15 x 10^-3^ |
| Glucocortocoid Receptor Signaling | 8.41 x 10^-3^ |
| IL-10 Signaling | 1.08 x 10^-2^ |
| Pentose Phosphate Pathway | 1.34 x 10^-2^ |
| Glutathione Metabolism | 1.43 x 10^-2^ |
| D-glutamine and D-glutamate Metabolism | 1.46 x 10^-2^ |
| Hypoxia Signaling | 1.88 x 10^-2^ |
| PPAR Signaling | 1.94 x 10^-2^ |
| Arginine and Purine Metabolism | 2.02 x 10^-2^ |
| Glutamate Metabolism | 2.04 x 10^-2^ |
| Role of Cytokine in Mediating Communication between Immune Cells | 2.2 x 10^-2^ |
| Aldosterone Signaling in Epithelial Cells | 2.26 x 10^-2^ |
| Cardiac Hypertrophy Signaling | 2.61 x 10^-2^ |
| Glycosphingolipid Biosynthesis- Neolactoseries | 3.07 x 10^-2^ |
| Role of BRCA1 in DNA Damage Response | 3.36 x 10^-2^ |
| Role of CHK Proteins in Cell Cycle Checkpoint Control | 3.89 x 10^-2^ |
